# Effects on health-related quality of life of interventions affecting survival in critically ill patients: a systematic review

**DOI:** 10.1186/s13054-022-03993-3

**Published:** 2022-05-06

**Authors:** Ottavia Pallanch, Alessandro Ortalda, Paolo Pelosi, Nicola Latronico, Chiara Sartini, Gaetano Lombardi, Cristiano Marchetti, Nicolò Maimeri, Alberto Zangrillo, Luca Cabrini

**Affiliations:** 1 Department of Anesthesia and Intensive Care, IRCCS San Raffaele Scientific Institute, Milan, Italy; 2 Department of Surgical Sciences and Integrated Diagnostic (DISC), University of Genoa, Viale Benedetto XV 6, Genoa, Italy; 3 Anesthesia and Intensive Care, San Martino Policlinico Hospital, IRCCS for Oncology and Neurosciences, Genoa, Italy; 4 Department of Medical and Surgical Specialties, Radiological Sciences and Public Health, University of Brescia, Brescia, Italy; 5 Department of Anesthesia, Intensive Care and Emergency, ASST Spedali Civili University Hospital, Piazzale Ospedali Civili, 1, Brescia, Italy; 6 “Alessandra Bono” University Research Center for LOng-Term Outcome (LOTO) in Survivors of Critical Illness, University of Brescia, Brescia, Italy; 7 ASST Sette Laghi, Ospedale di Circolo Fondazione Macchi, Varese, Italy; 8 Vita-Salute San Raffaele University, Milan, Italy; 9 Dipartimento di Biotecnologie e Scienze della Vita, Università degli Studi dell’insubria, Varese, Italy

**Keywords:** Critical care, Critical illness, Quality of life, Patient-important outcomes, Long-term outcomes, Mortality

## Abstract

Survival has been considered the cornerstone for clinical outcome evaluation in critically ill patients admitted to intensive care unit (ICU). There is evidence that ICU survivors commonly show impairments in long-term outcomes such as quality of life (QoL) considering them as the most relevant ones. In the last years, the concept of patient-important outcomes has been introduced and increasingly reported in peer-reviewed publications. In the present systematic review, we evaluated how many randomized controlled trials (RCTs) were conducted on critically ill patients and reporting a benefit on survival reported also data on QoL. All RCTs investigating nonsurgical interventions that significantly reduced mortality in critically ill patients were searched on MEDLINE/PubMed, Scopus and Embase from inception until August 2021. In a second stage, for all the included studies, the outcome QoL was investigated. The primary outcome was to evaluate how many RCTs analyzing interventions reducing mortality reported also data on QoL. The secondary endpoint was to investigate if QoL resulted improved, worsened or not modified. Data on QoL were reported as evaluated outcome in 7 of the 239 studies (2.9%). The tools to evaluate QoL and QoL time points were heterogeneous. Four interventions showed a significant impact on QoL: Two interventions improved survival and QoL (pravastatin in subarachnoid hemorrhage, dexmedetomidine in elderly patients after noncardiac surgery), while two interventions reduced mortality but negatively influenced QoL (caloric restriction in patients with refeeding syndrome and systematic ICU admission in elderly patients). In conclusion, only a minority of RCTs in which an intervention demonstrated to affect mortality in critically ill patients reported also data on QoL. Future research in critical care should include patient-important outcomes like QoL besides mortality. Data on this topic should be collected in conformity with PROs statement and core outcome sets to guarantee quality and comparability of results.

**Supplementary Information:**

The online version contains supplementary material available at 10.1186/s13054-022-03993-3.

## Background

In the last 50 years, the number of patients admitted to intensive care unit (ICU) has increased [1, 2]. This is particularly true after the SARS-CoV-2 pandemic [3]. At the same time, new technologies and improvements in critical care have positively influenced survival after ICU admission [4–6].

Survival is easy and reliable to be collected, and it has been traditionally considered as the most important outcome to be improved in ICU, both by clinicians and by researchers [7]; accordingly, the identification and implementation of the interventions improving survival in ICU is a crucial task investigated in many randomized control trials (RCTs) and systematic reviews [8–10].

Recently, the concept of patient-important (or patient-centered) outcomes, including long-term quality of life (QoL), functional, cognitive and mental health outcomes, has been introduced, to consider patients’ feelings and values in a holistic and comprehensive perspective [7, 11–13]. Moreover, there is evidence that patients discharged from ICU consider survival as one of the less important outcomes, while physical, cognitive and mental outcomes and the ability to return to work are perceived as the most relevant [7, 13]. ICU survivors commonly show impaired QoL [14, 15], defined from World Health Organization as “individual perceptions of their position in life in the context of the culture and value systems in which they live and in relation to their goals, expectations, standards and concerns” [16, 17]. Patient-important outcomes in ICU survivors have been increasingly reported in peer-reviewed publications in the last years, even if using heterogeneous instruments [18]. In the present systematic review, we evaluated how many randomized controlled studies were conducted on critically ill patients and reporting a benefit on survival also reported data on the treatment effect on QoL.

## Material and methods

In a first stage, all published RCTs investigating nonsurgical interventions that reduced mortality in critically ill patients were identified on MEDLINE/PubMed, Scopus and Embase. There was no time limit, and the date of last update was August 31, 2021. The full MEDLINE/PubMed search strategy was already published [8, 9] and is available in the Additional files (Additional file 1). We followed the Preferred Reporting Items for Systematic Reviews and Metaanalyses (PRISMA) guidelines and PRISMA checklist is included in Additional file 1 (Additional Table 1).

Inclusion criteria for the selected papers were: (1) be published in a peer-reviewed journal, (2) be classifiable as RCT, (3) investigating nonsurgical interventions (drug, technique or strategy), (4) involve critically ill patients, and (5) interventions should significantly reduce mortality.

Exclusion criteria were: (1) not RCTs (observational studies, quasi-randomized studies), (2) nonsignificant reduction in mortality before statistical adjustments, or reduction only in subgroups, (3) classification as surgical procedure, and (4) intraoperative intervention.

The definition of “critically ill patient” was determined by the presence of acute failure of at least 1 organ, ICU admission, or emergent treatment.

Only landmark mortality (evaluated at a specific time point), obtained without adjustment for baseline characteristics, was considered statistically significant when *p* < 0.05.

In a second stage, all the studies identified in the first stage were then screened by two independent reviewers to identify the presence of the outcome “quality of life,” according to the World Health Organization definition of QoL [16, 17].

Full-text articles were downloaded when the abstract lacked information and data on QoL were extracted in addition to those on mortality. The following data were collected: QoL p value, number of patients included in QoL analysis, QoL score or scale used, QoL time point, time frame and baseline, how QoL questionnaires were administered, and if the intervention of a proxy was admitted. Data were independently retrieved from the selected articles by two reviewers. Discrepancies were resolved by consensus or by a third reviewer.

## Results

**Fig. 1:**
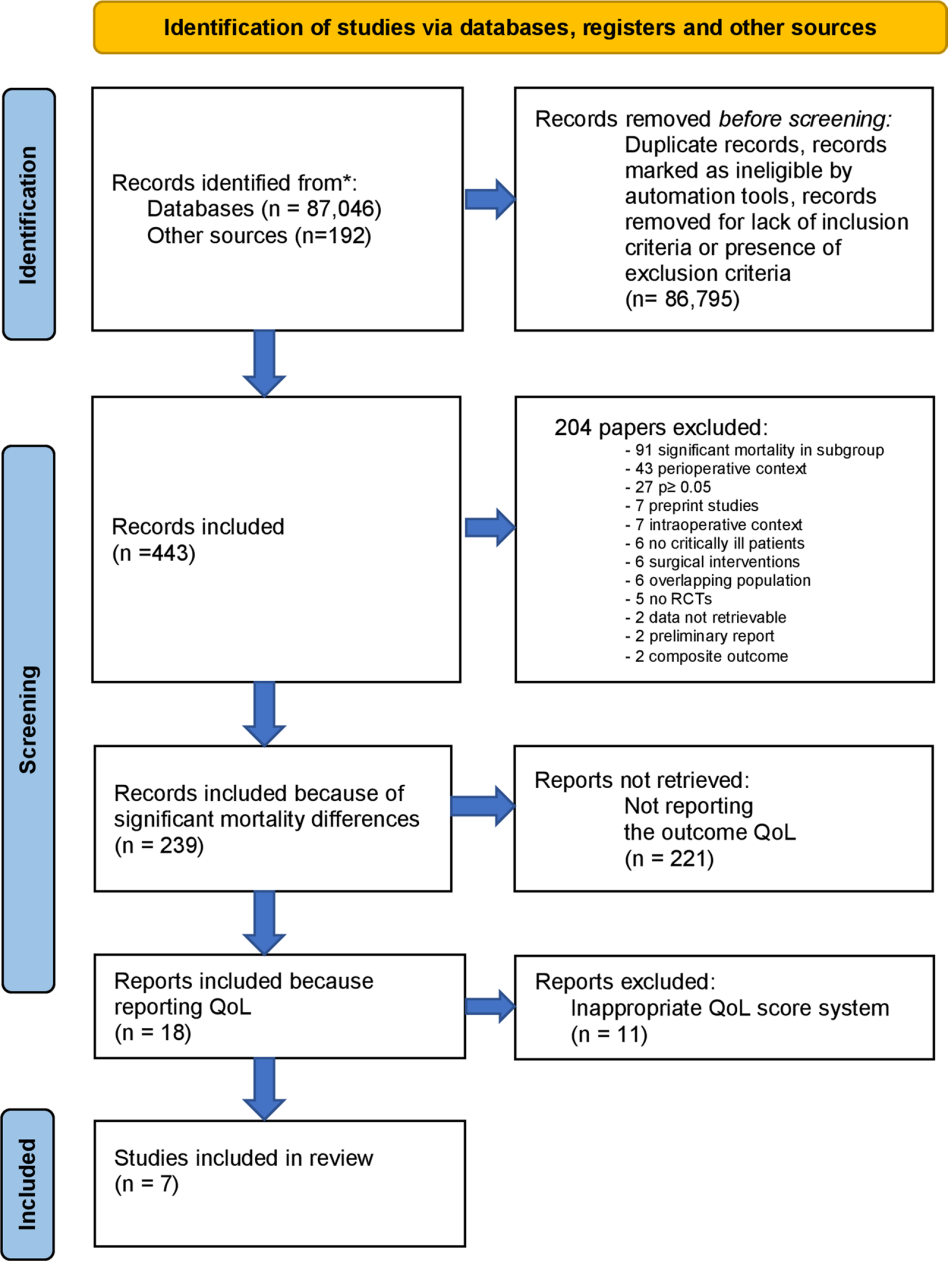
PRISMA 2020 flow diagram for systematic reviews. From: Page MJ, McKenzie JE, Bossuyt PM, et al. The PRISMA 2020 statement: an updated guideline for reporting systematic reviews. BMJ 2021;372: n71

We identified 239 RCTs performed in critically ill patients reporting a statistically significant reduction in mortality. Among these, 18 studies reported data on QoL and were initially selected. After checking the full texts, 11 articles were excluded for inadequacy of the QoL scores used. Seven out of 239 (2.9%) RCTs were included in final analysis [19–25], with an overall population of 7,696 patients (Tables 1, 2). The whole process of study selection is described in Fig. 1. Five studies were multicenter [20–23, 25] and three were multinational [20, 22, 23]. The longest follow-up with significant effect on mortality differed between studies and ranged between 7 days and 2 years. The outcome “quality of life” was reported for 5396 patients. Other 2214 patients died, and 86 patients were lost to follow-up before data on QoL were collected.

**Table 1:** Characteristics of the studies included

References	Topic	Population	Sample size	Intervention	Control	Mortality *p* value	Longest follow-up with significant mortality differences
Tseng et al. [19]	Pravastatin in subarachnoid hemorrhage	Aneurysmal subarachnoid hemorrhage patients (age 18–84 years, onset 1.8 ± 1.3 days)	80	Pravastatin 40 mg	Placebo	0.04	In-hospital mortality
Doig et al. [20]	Underfeeding in refeeding syndrome	Adults in ICU with refeeding syndrome	339	Caloric restriction	Standard caloric intake	0.04	90 days
Guidet et al. [21]	ICU triage	Critically ill patients aged 75 years or older who arrived at the ED	3037	Systematic ICU admission	Standard practice	< 0.01	180 days
Hanley et al. [22]	Thrombolytic removal of intraventricular hemorrhage in severe stroke	Patients with extra-ventricular drain, in the ICU with stable, non-traumatic intraventricular hemorrhage obstructing the 3rd or 4th ventricles	500	Up to 12 doses, 8 h apart, of 1 mg alteplase via extra-ventricular drain	Up to 12 doses, 8 h apart, of 0.9% saline via extra-ventricular drain	0.01	180 days
Sprigg et al. [23]	Tranexamic acid in hyperacute primary intracerebral hemorrhage	Adults with intracerebral hemorrhage from acute stroke units	2325	1 g intravenous tranexamic acid bolus followed by an 8-h infusion of 1 g tranexamic acid	Placebo same dilution and rate of infusion as treatment group	0.0406	7 days
Zhang et al. [24]	Dexmedetomidine after noncardiac surgery	Patients ≥ 65 years, admitted to ICU after noncardiac surgery	700	Dexmedetomidine loading dose of 0.6 µg/kg 10 min before anesthesia induction, then continuous infusion of 5 µg/kg/h until 1 h before the end of surgery	Normal saline same dilution and rate of infusion as treatment group	0.04	2 years
Parke et al. [25]	Fluid management after cardiac surgery	Adults undergoing elective cardiac surgery with CPB, with preoperative EuroSCORE II ≥ 0.9	715	Protocol-guided fluid administration, based on SVV, in ICU	Fluid management determined by local protocol and bedside clinician, in ICU	0.04	ICU discharge

ICU, intensive care unit; ED, emergency department; CPB, cardiopulmonary bypass; SVV, stroke volume variation

**Table 2:** QoL data of the included studies

References	Topic	Sample size for QoL analysis	QoL scale	QoL follow-up	QoL evaluation at baseline	How QoL questionnaires were administered	Proxies involved	Quality of life *p* value	Main findings
Tseng et al. [19]	Pravastatin in subarachnoid hemorrhage	60	SF 36	180 days	No	Direct interview	Not specified	< 0.01	Pravastatin reduces mortality and improves QoL
Doig et al. [20]	Underfeeding in refeeding syndrome	260	SF 36 general health and physical function domains	90 days	No	Not specified	Not specified	0.014	Caloric restriction improves overall survival but negatively affects QoL
Guidet et al. [21]	ICU triage	1763	SF 12	180 days	No	Not specified	Not specified	0.02	Systematic ICU admission reduces mortality but also reduces QoL in “mental” domain
Hanley et al. [22]	Thrombolytic removal of intraventricular hemorrhage in severe stroke	375	EQ-VAS/SIS	Not specified	No	Not specified	Not specified	0.37 EQ-VAS/0.31 SIS	Irrigation of the ventricles with alteplase via an extra-ventricular drain significantly improves survival, but don’t have significant impact on QoL
Sprigg et al. [23]	Tranexamic acid in hyperacute primary intracerebral hemorrhage	1808	EQ5D HUS/EQ5D VAS	90 days	No	Not specified	Not specified	EQ5D HUS 0.3/EQ5DVAS 0.13	Tranexamic acid significantly reduces mortality, but it does not affect QoL
Zhang et al. [24]	Dexmedetomidine after noncardiac surgery	434	WHOQOL-BREF	3 years	No	Telephone	Yes	< 0.0001	Dexmedetomidine improves survival and QoL
Parke et al. [25]	Fluid management after cardiac surgery	696	EQ 5D 5L	180 days	Yes	Not specified	Not specified	Not specified (0.023 only in pain/discomfort domain of the score)	Patients treated with a protocol-guided fluid management based on SVV after CPB had higher mortality without a significant impact on QoL

QOL, quality of life; Proxies, people who can make healthcare decisions on behalf of patients when they are no longer able to do it by themselves; SF-36 = RAND-36, Short Form 36; SF-12, Short Form 12; ICU, intensive care unit; EQ 5D 5L, EuroQoL 5D 5L; EQ VAS, EuroQol Visual Analogue Scale; SIS, Stroke Impact Scale; EQ HUS, EuroQoL Health Utility Score; WHOQOL-BREF, World Health Organization Quality of Life-BREF; SVV, stroke volume variation; CPB, cardiopulmonary bypass

The instruments used to assess QoL differed between included studies: Two studies used the Short Form 36 questionnaire (SF 36) [19, 20], one study used the Short Form 12 (SF 12) [21], one study used the World Health Organization Quality of Life-BREF (WHOQOL-BREF) [24], three studies applied Euro Quality of Life questionnaires in different forms: EQ 5D 5L, EQ VAS and EQ HUS [22, 23, 25]. One study treating severe stroke also used the specific Stroke Impact Scale in addition to the EQ VAS [22]. Only two of seven studies reported how the questionnaires were administered to patients: by telephone (one study) [24] or direct interview (one study) [19]. The longest follow-up for assessment of QoL was 3 months for two studies [20, 23], 6 months for three studies [19, 21, 25] and 3 years in one study [24]. In one study, the length of follow-up was not specified [22]. Patients’ baseline QoL was registered only in one study [25].

Four interventions showed a significant impact on QoL beside that on survival [19–21, 24]. Of these, two interventions consensually improved survival and QoL [19, 24], while two interventions reduced mortality but negatively influenced QoL [20, 21]. Three interventions did not significantly affect QoL [22, 23, 25]. Pravastatin in subarachnoid hemorrhage and dexmedetomidine in elderly patients admitted to the ICU after noncardiac surgery were effective both in reducing mortality and in improving QoL [19, 24]. Of the two interventions with opposite effects on survival and QoL, caloric restriction improved overall survival in patients with refeeding syndrome but had a negative effect on QoL in the general health domain of SF 36 questionnaire (*p* 0.014) [20]; the systematic ICU admission of elderly patients in place of usual triage improved survival but reduced QoL, specifically in mental aspects of QoL evaluated with SF 12 questionnaire (*p* < 0.01) [21].

## Discussion

The present study is the first to evaluate the impact on QoL of the interventions which showed a significant effect on survival in critically ill patients. We found that: (1) only 7 (2.9%) studies assessed QoL among the 239 RCTs reporting a significant effect on survival; (2) interventions improving survival can have not only a positive or null but also a negative effect on QoL.

Our findings raise the ethical need to establish if the improvement in survival outweighs the negative effect on QoL. Ideally, the single patient should be the judge: Therapeutic decisions should be shared with every patient, or, when not feasible, with his/her family and loved ones, addressing the expected impact of interventions not only on survival but also on QoL.

ICU survivors suffer from medium- and long-term impairments in mental health, cognition and physical function, because of premorbid conditions, severity of the acute illness and complications associated with treatments during the ICU stay [13, 26–33]. Only in 2010, the set of all these impairments was defined “Post-Intensive Care Syndrome” [6, 34, 35]. Implications of PICS are multidimensional and touch not only patients but also caregivers and society [36]*.* For all these aspects described above, long-term QoL is compromised among ICU survivors when compared with general population [14, 15].

Recent publications established the importance of a “patient-centered” approach in evaluating the impact of critical illness, and the relevance of considering also “patient-oriented” outcomes (besides “disease-oriented” outcomes) when conducting or evaluating a study [37–39]. Despite this, Gaudry et al. (in a study not focused on RCTs in which a significant effect on survival was present, in contrast to our study) found that only 10% of 112 RCTs published in 2013 including critically ill patients recorded at least one QoL functional/cognitive/neurological outcome assessed after ICU discharge [12].

In a review on post-discharge ICU outcomes, Dinglas et al. [7] pointed out that the heterogeneity in outcomes assessment may represent a major limitation of research in this field, making difficult the comparison across studies and metanalyses. Several proposals have been published to standardize QoL score systems and time to follow up. In 2018, an evidence-based extension of the SPIRIT 2013 statement was published. The aim was to identify additional patient-reported outcomes (PRO) items recommended for inclusion in clinical trial protocols (extensions) and to adapt the existing SPIRIT 2013 statement specifically as applied to PROs [40]. Moreover, completed core outcome sets already exist for studies on critical illness, which include QoL evaluation [41]. Assessment of QoL requires a multidimensional approach that includes “individual’s perception of their position in life in the context of the culture and value systems in which they live and in relation to their goals, expectations, and standards” [16, 17]. As such, QoL assessment requires multiple measures to fully consider subjectivity and multidimensionality, and it is of paramount importance that only validated instruments such as SF-36 or EQ-5D are used [42]. Despite this, at present, great variety persists in studies analyzing QoL [39, 41, 43].

In our work, even if EQ 5D 5L and SF 36 were the mainly applied scores, other scores or variants of these two were used in 4 out of the 7 included studies [21–24]*.* In addition, there were a variety of different time points for the follow-up.

In the present systematic review, we found that only two interventions showed a significant positive influence on both survival and QoL: pravastatin in subarachnoid hemorrhage [19] and dexmedetomidine in ICU after noncardiac surgery in patients aged more than 65 years old [24]. At the same time, it is important to note that other interventions, in particular caloric restriction in patients with refeeding syndrome [20] and systematic ICU admission of elderly patients arriving to the emergency department [21], can improve survival after critical illness, but reduce QoL at, respectively, 3 and 6 months. These findings may be also attributable to the fact that more patients in the treatment groups survived and could be assessed at follow-up for longer periods. Moreover, in those two studies the observed statistically significant difference in QoL was limited to one single QoL domain (RAND-36 general health in Doig [20] and SF-12 mental component score in Guidet [21]) and it was not considered to reach the minimum clinically relevant difference by the authors.

Even if the interest on this topic is growing, our review shows that to the present day little information is available on QoL after ICU discharge and this information is difficult to interpret because of the absence of standardization of data collection in this field.

Several barriers can explain the very limited exploration of QoL in ICU survivors: Long-term follow-up of survivors requires human and logistical resources, increasing the costs of a study [12]. Lengthy questionnaires can be required, but they are time-consuming, and this often increases the percentage of non-respondents [12]. Moreover, cognitive and communication difficulties and the need for trained interviewers are further limitations to inclusion of long-term QoL data in studies [12, 44].

Nevertheless, our review suggests that patients’ priorities are fundamental in daily clinical practice and in choosing the appropriate treatments, as patients could refuse a treatment with the potential of improving survival but significantly worsening the QoL, eventually preferring a treatment aiming at improving the quality of dying [12]. Thus, future research in critical care should aim to investigate effects of interventions on QoL in conformity with PRO statement and core outcome sets [40, 41].

Our study has some limitations. First, the included studies were markedly heterogeneous in their background: Different numbers of centers and patients, methods of QoL assessment and particularly the use of a phone or direct interview questionnaires make hard to compare the results. Second, we excluded all the non-RCTs and all the RCTs with nonsignificant effect on mortality, and then we did not evaluate all the studies reporting positive or negative effects on QoL. Third, we focused on the outcome “quality of life” according to a precise definition [16, 17], excluding other long-term outcomes and patient-important outcomes. We aimed to restrict to an outcome which was multidimensional and directly connected with patients’ self-perception of life, because patient involvement was of primary interest in our study.

## Conclusion

A minority of RCTs in which an intervention demonstrated to affect mortality in critically ill patients reported also data on QoL. Future research in critical care should include patient-important outcomes like QoL besides mortality. Data on this topic should be collected in conformity with PROs statement and core outcome sets to guarantee quality and comparability of results.

## Supplementary Information

**Additional file 1.**. Additional File 1 - Search string. Additional Table 1 - PRISMA 2020 Checklist

## Data Availability

The datasets used and/or analyzed during the current study are available from the corresponding author on reasonable request.

## Abbreviations

ICU: Intensive care unit
QoL: Quality of life
RCTs: Randomized controlled trials
PICS: Post-intensive care syndrome
SF-36 = RAND-36: Short Form 36
SF-12: Short Form 12
WHOQOL-BREF: World Health Organization Quality of Life-BREF
EQ 5D 5L: EuroQoL 5D 5L
EQ VAS: EuroQoL Visual Analogue Scale
EQ HUS: EuroQoL Health Utility Score
SPIRIT: Standard Protocol Items: Recommendations for Interventional Trials
PROs: Patient-reported outcomes
